# Rethinking Remote Work, Automated Technologies, Meaningful Work and the Future of Work: Making a Case for Relationality

**DOI:** 10.1007/s13347-023-00634-7

**Published:** 2023-04-28

**Authors:** Edmund Terem Ugar

**Affiliations:** 1 Department of Philosophy, University of Johannesburg, Johannesburg, South Africa; 2 Centre for the Philosophy of Medicine, Public Health, and Epidemiology, University of Johannesburg, Johannesburg, South Africa; 3 Centre for the Philosophy of Medicine, Public Health, and Epidemiology, Durham University, Durham, UK

**Keywords:** Remote work, Interpersonal relationship, Collegial relationship, Sub-Saharan Africa, Meaningful work

## Abstract

Remote work, understood here as a working environment different from the traditional office working space, is a phenomenon that has existed for many years. In the past, workers voluntarily opted, when they were allowed to, to work remotely rather than commuting to their traditional work environment. However, with the emergence of the global pandemic (corona virus-COVID-19), people were forced to work remotely to mitigate the spread of the virus. Consequently, researchers have identified some benefits and adverse effects of remote work, especially in the age of COVID-19, ranging from flexible time and environment to technostress and isolation. In this paper, using a phenomenological approach, specifically, the sub-Saharan African experiences, I contend that remote work in the age of advanced technologies has obscured the value of relationality due to the problem of isolation in sub-Saharan African workplaces. For sub-Saharan Africans, relationality is a prerequisite moral value to becoming a person. In addition, relationality contributes to meaningfulness in the workspace. Obscuring the value of relationality in the aforementioned locale leads to meaninglessness in the workspace. Furthermore, this paper contributes to the existing literature on meaningful work by critically showing the importance of the value of relationality as a key element that adds meaning to work in sub-Saharan Africa.

## Introduction

The proliferation of disruptive technologies,^1^ such as information technology (IT), has warranted scholars like Mueller (2021) and Jones (2021) to advance a view that these ubiquitous technologies may lead to sporadic unemployment in the near future. Contrary to the above view, other theorists like Bastani (2019) and Danaher (2019) argue that introducing sociotechnical systems,^2^ such as artificial intelligence, robots, and information technology, in the workspace may only lead to eradicating meaningless work. However, the rampant advancement and introduction of automated technologies are currently forcing humans to reconsider what they take to be work or meaningful work.

I take the most simplistic definition of work to mean paid employment or activities comparable (not paid for but have the potential to be paid for) to paid employment even though they are not paid for. The Oxford English Dictionary defines work in two ways: first, work means “action or activity involving physical or mental effort and undertaken in order to achieve a result esp. as means of making one’s living or earning money; labour; (one’s) regular occupation or employment” (Oxford English Dictionary, 2022). This definition points to the view that work is generally a person’s job or whatever one does that takes up the person’s effort and time. Despite the view that work is an activity done by a person to earn a livelihood, some thinkers argue that work has meaning (Lysova et al., 2019; Smids et al., 2020; Jones, 2021). However, these views are not necessarily conflicting as work can be considered a means of livelihood and still be meaningful.

Meaningful work is the benefits work brings to human lives besides the socio-economic importance of work. Meaningful work is the significance of work to the human person (Lysova et al., 2019). Work, in this sense, is considered meaningful because it is important for the worker’s good. Nicolas Carr (2014, 2016) contends that removing paid jobs or giving them to machines poses adverse effects as it affects the very essence of human life and the meaningfulness of working. Furthermore, Carr (2016) argues that automating jobs does not eliminate hardship but removes what is fundamentally valuable to humans. However, with the exponential increase in technology and the forceful move to remote work, there has been a challenge to meaningful work.

Remote work, a type of work that is done outside the traditional working environment, such as offices to homes, has been in existence since the beginning of the Industrial Revolution (Jumbo, 2022). However, before the emergence of the global pandemic (COVID-19), remote work was a working condition workers opted for when allowed to; it was not compulsory. However, COVID-19 changed the notion of remote work, making it mandatory for almost everyone to work remotely. Craig and Churchill (2021), Bin et al. (2021), and Akuoko et al. (2021) argue that the beginning of the hard lockdown in some countries led to work intensification, surveillance and monitoring of employees by their employers, working for longer hours, combining family time and work time together, and isolation and loss of relationships in the workplace. However, while the rest of these issues are sufficiently discussed in the current literature (see Carillo et al., 2021; Burk et al. 2021; Ipsen et al., 2021), the issue of isolation and loss of relationships in the workplace caused by remote work has not been sufficiently explored.

Bin et al. (2021) and Carillo et al. (2021) passively addressed the issue of isolation and loss of relationships caused by remote work. Thus, in this paper, I contend that the absence of sufficient human–human relationships in remote work environments causes isolation. In some contexts, like sub-Saharan Africa, the value of human–human relationships/relationality is an important moral value and a vital contributor to meaningful work. A workplace, for sub-Saharan Africans, is an environment where interpersonal relationships are created, besides the economic remuneration, since interpersonal relationships shape workers’ personhood (Baloyi & Makobe-Rabotha, 2014:233; Metz, 2022). Interpersonal relationship in the work environment goes concomitantly with economic remuneration to result in meaningful work.

Given the above contention, this paper contributes to the discourse on meaningful work and the future of work by bringing to light two major elements that risk meaningful work in sub-Saharan Africa: automated technology and remote work. I argue that in a highly technological age where automated technologies are proliferating, and remote work has formed an integral aspect of work, there have been more experiences of isolation and loss of relationships within the workspace. On the one hand, the feeling of isolation stems from the lack of relationality with other human subjects within the workplace. On the other hand, relationality is an important aspect of the work environment in sub-Saharan Africa due to the meaning relationality adds to work.^3^ Given these problems, it follows that automated technologies in conjunction with remote work obscure the meaning of work and threaten the future of work as a meaningful endeavour in sub-Saharan Africa.

I structure this paper as follows: In the first section, I give an exposition of what work entails. Afterwards, I show the value of work by engaging with the notion of meaningful work in the second section. In the third section, I ask whether remote work contributes to the meaning of work within the sub-Saharan space. In the fourth and fifth sections, I show the influence of technologies, such as information technology (IT), artificial intelligence (AI), and robots, within the remote work setting. Furthermore, I show how these technologies both actively and passively obscure the value of relationality within the workspace, with a particular interest in the sub-Saharan African workspace. In addition, I discuss the notion of isolation caused by technologies as a major deterrent to relationality in the workplace. In the sixth section, I ask whether technologies like robots and AI can fill the lacunas created by remote work, such as collegial relationships, and whether the relationship with robots has moral value for sub-Saharan Africans in terms of strengthening their personhood. In the last section, I discuss some possible objections to my argument and respond to them.

## An Exposition of the Meaning of Work

The definition of work has two assumptions, the conventional assumption and the expanded conventional assumption^4^ (Eichler & Mathews, 2004). The conventional view holds the claim that work is anything that is paid for, and activities that are not paid for are not regarded as work (Eichler & Mathews, 2004). In my view, activities are generally defined as work if the activities are directed towards achieving some goals, such as payments. However, this view can be slightly contested, as one might say that the goal of activities is not payment. Rather, payment is just a reward given after achieving the goal. My point here is that it almost goes without saying that, in most cases, we perform such activities because we want to be paid. For instance, some employers in “toxic” environments say things like, “I am only taking this nonsense in this workplace because I need the money to take care of my bills.” This shows that they work because of the remuneration they get. Furthermore, work is whatever activity that requires energy expenditure, but it must be for a specific goal, such as earning a living, and not for the fun of exacting energy (Eichler & Mathews, 2004:14–5).

For an activity to be considered as work, the activity must include the expenditure of energy with a specific goal orientation rather than for the sake of working. This definition excludes activities undertaken for the fun of the activity or somewhat involuntary activities, such as sleeping and breathing. For an activity to be considered as work, the activity will depend on the occasion on which this activity is undertaken. For example, if we watch soccer for the fun of watching soccer, then such an activity cannot be called work. However, if we watch soccer to coach a team or analyse the game for payment, that becomes work even though the activity could be fun-filled (Eichler & Mathews, 2004:19).

The conventional meaning of work can be traced to the works of Max Weber in his seminal book *The Protestant Ethic and the Spirit of Capitalism* (1905), which engages the understanding of the conventional notion of work. Although Weber uses the concept of labour, I think we can use labour and work interchangeably in this context since they involve the exertion of energy for payment. As a result, in this paper, I will use work in place of labour. In Karl Marx’s work, some elements of the conventional notion of work involve transforming the natural world, leading to the materialisation and actualisation of human needs (Marx, 1867 [2004]). In this sense, work became an important aspect of human existence (Engels, 1876 [1950]); therefore, work has become “a social value and organising principles of modern ‘material life’” (Just, 2014:446).

In their works, Marx, Hegel, and Weber attached some meaning to their understanding of work. They see work as self-realisation, that is, work allows workers to express their selfhood. On the contrary, Graeber (2018) and Harding (2019) argue that, in the contemporary understanding of work, the only value that is attached to work is the economic gains of working since many jobs only offer wages to workers and nothing more in our current social milieu. However, Smids et al. (2020) argue that work has meaning beyond economic gains. In what follows, I engage with what has been theorised as meaningful work in the literature on work.

## Meaningful Work: An Exposition of the Literature

The conventional meaning of work makes some contemporary work meaningless and some meaningful, the same way some jobs are paid and others are unpaid. Some conventional work brings meaning beyond their economic gain (O’Brien, 1996). These jobs provide meaning by carving some opportunity to express one’s identity and autonomy. Furthermore, these jobs allow workers to participate in a meaningful cause (Hofmeister, 2019). As a result, they can be considered meaningful jobs.

Carr (2014, 2016) contends that paid jobs are the only jobs that are meaningful, and the removal of these kinds of jobs will result in the meaninglessness of work. Paid work contributes to the essence of human life, and the removal of paid jobs can bring the notion of work to meaninglessness (Carr, 2014, 2016). However, Eubanks (2019) and Jones (2021) argue that the future of work will be the complete removal of paid work due to the proliferation of disruptive technologies. As a result, there is a possibility that the proliferation of disruptive technologies might result in the elimination of hardship as well as meaningfulness to humans, as the most fundamental essence of life, which is work, is distorted^5^ (Carr, 2016; Lysova et al., 2019; Smids et al., 2020; Jones, 2021).

However, it could also be argued that technologies come with the solution to many existent issues caused by work in different societies. For instance, if all paid jobs are automated, this may lead to humans having more free time to engage in other non-work activities. In this sense, disruptive technologies present an encompassing advantage to different workspaces, especially when removing meaningless, boring, and menial work, which then opportune humans to spend more time doing meaningful things they see fit. Furthermore, proponents of post-work, such as Jones and Winder (2021) and Alexander (2020), allude that with the existence of automated technologies such as AI, humans can now have quality time doing what they deem necessary and things that align with their meaningful goals. As a result, post-work proponents call for the transfer of work to AI systems (Jones & Winder, 2021; Alexander, 2020).

With automated technologies, Bastani (2019) and Danaher (2019) hold that technologies may lead to the utopian emancipation of our societies from meaningless work. However, Jones (2021) and Mueller (2021) contend that the proliferation of technology may lead to unintended technological unemployment and the threat of disruptive technologies to the work sector. As a result, this current disruptive technological movement in the workspace forces humans to reconsider what work means. This is because the ubiquity of social technology, such as AI, poses a significant crisis in the traditional notion of work, eliminating work, or making work meaningless and inhuman. The future of work is that which is technologically oriented, given advanced disruptive technological innovations in our different societies currently. These recent innovations in the workspace, especially regarding the automation of jobs, leave human workers somewhat obsolete or less involved.^6^

However, Smids et al. (2020) point out that doing work comes with meaning, and work should not be completely transferred to AI technology. This is because meaningful work involves engaging with work beyond the biological necessity of work (Steger, 2019). The notion of meaningful work has been an area of discussion that has been tremendously spiked by the emergence of the COVID-19 pandemic, which resulted in many countries engaging in total lockdown measures. The meaningfulness of work has become more pressing in this social milieu than in the past years (Lipman, 2021; Savage, 2020). Even though people still strive to get meaning in their work, for the most part, meaningful work still revolves around economic remuneration (Keohane, 2015). Here, meaningful work has to do with a person’s engagement in an activity in ways that express their personhood, value, and idea. I return to this discussion in the next section.

For work to be meaningful, the work has to be worthwhile and must have some significance and relevant good (Lysova et al., 2019). The meaning of work is best fostered when a work environment creates the opportunity for individuals to find meaning in their work. Individuals spend a considerable amount of time and energy working; as a result, it matters that their work is accorded meaning besides the economic remuneration. Furthermore, the meaning of work should be a matter of justice (Gheaus & Herzog, 2016).

However, since the meaning of work is that which has considerable good and is worthwhile, the problem that arises from this definition is the question of how we measure what is worthwhile and good. Scholars from different disciplines engaging with the meaning of work, from business ethics and sociology to political philosophy, have yet to agree on what constitutes the meaning of work (Lips-Wiersma et al., 2018; Bailey et al., 2019). Nonetheless, Smids et al. (2020) provide what they consider the most important aspect of what makes work worthwhile and good.

According to Smids et al. (2020), the core of meaningful work includes autonomy, pursuing a purpose, social relationships, self-esteem and recognition, and self-development and exercising skills. For Smids et al. (2020), to find meaning in work, one must develop themselves by exercising their skills, having a social bond with co-workers, having a purpose and pursuing the purpose, enjoying some form of autonomy, and receiving recognition at work.

Finding meaning in work is juxtaposed with finding meaning in life. In most instances, the meaning in life is judged by the pursuit of purpose in life (Baumeister & Vohs, 2002). Likewise, the meaning of work is seen as finding purpose in the wider community of one’s working environment (Wolf et al., 2010). According to some work psychologists, like Pratt and Ashforth (2003), Grint (1998), and Martela and Pessi (2018), striving to find purpose in work makes work meaningful. In this way, what could be considered objectively meaningful work is the ability of workers to find a purpose for themselves or others in the workspace (Veltman, 2016).

According to the five essences of work, as exposed by Smids et al. (2020), work as a place for exercising skills is a place to develop oneself, given the considerable time people spend at work. The skills one gain from the jobs result from the time one spends on the same job, solving complex issues, gaining specialised skills, and gaining some work virtues. Exercising these skills and developing oneself are necessary for finding meaning at work (Gheaus & Herzog, 2016; Lysova et al., 2019).

Second, autonomy at work means the ability for one to exercise their capacity of judgment and decision-making when provided sufficient discretionary room. Bowie (1998) and Roessler (2012) argue that there is a relationship between autonomy and meaningful work. For the above theorists, the relationship between autonomy and meaningful work relates to modifying tasks, interaction, and how one views their work. Some psychologists like Ward and King (2017), and Lysova et al. (2019) also argue that exercising one’s autonomy at work adds to meaningful work. Autonomy at the workplace allows workers to exercise their decision-making and display understanding at work. The next key essence of meaningful work is social relations at the workplace.

Exercising social relations at the workplace is what I consider to be the most important aspect of meaningful work beyond the economic remuneration, especially for people of sub-Saharan African descent. As stated earlier, this does not imply that social relations are not important to others beyond sub-Saharan Africa. It is more important to sub-Saharan Africans because participating in social relationships is an important requirement for moral excellence. For an individual to be considered as a person, the individual must create as many social networks as possible, and creating social networks in the workplace is a very important one.

For Madden and Bailey (2016) and Ward and King (2017), meaningful work is characterised by social relationship-building at the workplace by individuals. The social relationship is necessary for the human need to belong to a particular group dynamic; working with a community of other workers creates an environment of shared identity, agency, and purpose (Lysova et al., 2019; Rosso et al., 2010), especially for sub-Saharan Africans. Suppose a work environment does not enable the occasional fostering of social relationships between their workers; in that case, it affects their personhood, especially in contexts that consider social relationships an important moral requirement for moral excellence, such as in sub-Saharan Africa. In what follows, I show the importance of social relationships in the workspace within the sub-Saharan African context.

## The Workspace in Sub-Saharan Africa: An Environment for Interpersonal Relationships

The recurrent worldview in sub-Saharan Africa is the idea of community-centredness and interpersonal relationships (Horsthemke, 2015; Etieyibo, 2017; Metz, 2011, 2015). According to Metz (2015), sub-Saharan Africans consider communal relationships to be the most important aspect of their society over everything else. The idea of what it means to be in a communal relationship for Africans is clearly espoused by sub-Saharan African philosophers such as Mbiti (1970), Gyekye (1996), Wiredu (1996), Tutu (1999), Molefe (2017), Ikuenobe (2016), and Metz (2022), to mention a few. However, I will expose some of these ideas soon. In the meantime, the importance of communal relationship as briefly pointed out before, is that communal relationship is a prerequisite for moral excellence and the conferment of personhood for a sub-Saharan African.

In brief, personhood is very important in African philosophical discourses because personhood is considered the climax “of an African difference in philosophical theory” (Masolo, 2010:135). In sub-Saharan Africa, personhood is defined based on the combination of some normative and ontological criteria (Wiredu, 1996:2009). The ontological criterion is that, for one to become a person, the entity has to belong to the human species by possessing what constitutes human nature (Gyekye, 1996). However, being a human being is not enough; the individual has to meet some normative requirements, such as communal recognition based on the individual’s moral actions (Ikuenobe, 2016; Menkiti, 1984; Metz, 2022; Molefe, 2017; Ugar, 2022; Wiredu, 1996). These moral actions include, but are not limited to, participating in the activities of the community, participating in the rites of initiation/rites of passage, and building interpersonal relationships with other members of the community. In this paper, I focus on the aspect of individuals building interpersonal relationships as a prerequisite for moral excellence and personhood. This is because, in my view, the relational criterion for personhood is the most important requirement for moral excellence, and the technological-driven remote work arrangement threatens it.

In sub-Saharan ethics, an individual can only be called a person if they have shown more strides to be in a community through their relationship with others. This is because an individual can only be considered a person by others. In other words, “a person is a person through other people” (Mbiti, 1970:141). A person is one who is a member of a community rather than some abstract entity that is characterised by some metaphysical qualities such as autonomy, free will, or rationality (Menkiti, 1984). For an individual to be considered a person, they must be a community member and participate in the responsibilities that accompany their membership in the community (Ikuenobe, 2016). In what follows, I show what sub-Saharan Africans consider communal relationships and what living in a community with others means.

Sub-Saharan Africans consider communal relationships and community living as one which ought to be guided by harmony. Those who live in a community must see themselves as sharing the same identity and be prepared to express solidarity with each other (Metz, 2022). Metz’s (2022) conception of interpersonal relationships, which I prize, is one which is informed by values, such as identity and solidarity. This idea of identity and solidarity draws from the African value of harmonious relationships, which theorists like Mbiti (1970), Mokgoro (1998), and Tutu (1999) espouse. For example, Mbiti makes this point clear when he says, “I am because you are since you are, I am” or “a person is a person through other people” (Mbiti, 1970:141). In addition, Mokgoro points out that harmony means relating sympathetically with members of our social group (Mokgoro, 1998:17). While Tutu theorises that living in harmony is having the capacity to belong, share, and participate in a particular way of life with members of our social group (Tutu, 1999:35).

Metz (2022) outlines two ways to belong, participate, and share a way of life with others. First, members of the group must see themselves as sharing the same identity. In this sense, they must consider themselves as “part of the whole, being close, participating, sharing a way of life, belonging, and thinking of oneself as bound to others” (Metz, 2022:147). They must regard themselves as we, rather than I; they must think of themselves as common members of the group with the others (Metz, 2022:149). In addition, they must not feel isolated from the group or let others feel isolated from their presence (2022:149). Finally, they must cooperate with others, not on a prudential rationale, but for their own sake, to achieve their goals (2022:149).

Second, members of the community must exhibit solidarity with each other. They must do this by means of “achieving the good of all, being sympathetic, sharing, promoting the common good, engaging in service, and being committed to others’ good…caring for others’ quality of life” (Metz, 2022:147). To be in solidarity with other members of the community, one must be able to sympathise and empathise with others by knowing how it feels to be the other. Those in the above social relationships must strive to improve the quality of life of the other and advance the self-interest and self-realisation of others.

These criteria should be met for one to be considered as being in a communal relationship and living in a community within the sub-Saharan African framework. It is in exhibiting or having the capacity to exhibit identity and solidarity, as exposed here, that makes one has the moral status of personhood; as a result, being considered as a moral subject and object (Metz, 2022:168).

The sub-Saharan African relational African moral theory, espoused by Metz (2022), which is based on the friendly ways of relating with members of the community through identifying with them and being in solidarity with them, is deontological rather than teleological. It is based on the duties we owe each other in the web of relationships between subjects and objects in a communal setting. This idea of communal relationships cuts across every aspect of African society, which also gives meaning to work. This is because the meaning of work in sub-Saharan Africa goes beyond economic remuneration (Baloyi & Makobe-Rabotha, 2014). Work has meaning as long as it provides an environment for workers to accrue qualities, such as creating social and interpersonal relationships to enable them to become more persons in the workplace.^7^ This is because, as mentioned earlier, we owe each other interpersonal relationships in the workplace and the wider society in sub-Saharan Africa.

Sub-Saharan Africans consider the workplace as an avenue to foster interpersonal relationships and, as a result, create meaning in their work (Baloyi & Makobe-Rabotha, 2014:233). It is a prerequisite for the work environment to be an environment that fosters relationships to enable their employees to thrive morally by way of relating with each other through identity and solidarity (Baloyi & Makobe-Rabotha, 2014:233; Metz, 2022). Given the importance of social relationships in the workspace for Africans to have meaningful work, I now engage with the implications of automated technology and remote work that have increased at a high rate and pace given the emergence of the COVID-19 pandemic. In the next section, I argue that automated technologies and remote work, which are major characteristics of the future of work, threaten what is considered meaningful work for sub-Saharan Africans as it raises the problem of isolation.

## The Implications of Automated Technology and Remote Work to Social Relationships: A Necessity for Meaningful Work in Sub-Saharan Africa

In the previous sections, I explained what the concept of work means and what it means to engage in meaningful work. I exposed that meaningful work is underpinned by some essences ranging from autonomy to social relationships in the workplace besides the economic gains of work. I further showed that in sub-Saharan Africa, work is meaningful if workplaces create and facilitate opportunities for people to relate with each other in friendly ways as those who consider themselves as sharing the same identity and solidarity. Interpersonal relationships are integral moral requirements for moral status in sub-Saharan Africa. In this section, I look at ways remote work and automated technologies affect the value of interpersonal relationships in the workplace. I begin by defining what I mean by remote work.

Remote work, as mentioned earlier, is a work arrangement that has existed for quite some time, especially during the dawn of the Industrial Revolution. Remote work, also known as telecommuting, is the option that is availed to workers to have a flexible work arrangement to work from home or any space the worker finds convenient, using technologies such as the internet, telephone, cell phone, laptops, and other information and communication technologies (Allen et al., 2013; Gajendran & Harrison, 2007; Singh et al., 2017). Technological innovations have driven remote work with developments such as satellite offices, client offices, and telecentres (Flores, 2019). The current workforce is that which is technologically oriented with major innovations in the technological industries. With the advancement of sophisticated disruptive technologies, workers have opted, in most cases, to work remotely using available sociotechnical systems where the opportunities avail themselves. This arrangement implies that workers do not need to commute to work as they usually do if the option is unavailable. As a result, before the beginning of the COVID-19 pandemic, remote work was considered in some contexts as a flexible, technologically feasible, family-friendly work arrangement.

In some instances, remote work options have been provided by firms as a way to attract competition and enhance their employees’ work-life (Morgan, 2014; Felstead & Henseke, 2017). In this way, employers were willing to alter their work environment to fit the needs of their employees. Furthermore, such a move was a way of creating a work-family balance for employees (Hyland et al., 2005).

However, despite some workers’ voluntary opt-in to remote work, the recent COVID-19 pandemic, which brought about an involuntary remote working environment, has changed how remote work is visualised (Carillo et al., 2021). The switch to remote work from the traditional working environment was abrupt; as a result, some people were not prepared for the switch (Bin et al., 2021). Many workers had to learn how to use new technologies to work efficiently (Bin et al., 2021), especially in sub-Saharan Africa, given the relatively fair technological literacy in the continent. While some workers adjusted and appreciated the remote work environment as it allowed them to have a work-family balance and optimise their work performance (Carillo et al., 2021; Burk et al., 2021). Some other workers faced adverse effects of the remote working environment, such as isolation and work intensification (Akuoko et al., 2021; Bin et al., 2021; Craig & Churchill, 2021). However, this paper focuses on the adverse effects of remote work, such as isolation and its implication for sub-Saharan Africans.

In the social sense, isolation has been an area of sociological scholarship for a very long time, with the works of Durkheim (1997 [1897]), Max (1867 [2004]), and Williams (1987). In the studies of the theorists mentioned above on social isolation, they used concepts such as “alienation,” in the case of Max, and the “unattached” to discuss this concept. Social isolation has to do with a person’s inadequacies to engage or involve themselves in social relations and connect with other people in smaller and wider social groups and society. Social isolation shows that an individual is unable to get involved in interpersonal relationships (Masoom, 2016).

In their studies, Gartly Jaco (see Masoom, 2016) offers criteria for social isolation, of which two factors/criteria apply to this paper, even though Jaco’s study is relatively old. These criteria are anonymity, the remote location of friends, low occupational participation, high spatial mobility, low frequency of participation in groups and institutions, and low frequency of participation with other communities. I think the criteria important to this paper are remote locations of friends, and low participation in groups and institutions. I argue that with the advancement of technologies and the creation of remote working environments, there is a limitation these technologies and the environment poses for people to interact as they would if these technologies or remote working environment were not present. The combination of these technologies and remote work aggravates social isolation, which I discuss here.

For example, in the context of sub-Saharan Africa, on the one hand, the use of information and communication technology and the enforced remote workspace, which is now an aspect of the current workforce and the future of work, obscures the value of relationality, an important criterion for moral excellence to sub-Saharan Africans in the following ways. First, information technologies are currently used as automating technologies in the workspace. For example, using Microsoft Teams and Zoom, which is now slowly becoming a norm in sub-Saharan African countries, obscures more social relations between workers. With Zoom and Teams, there are less social relationships that exist with workers compared to when humans had contact meetings at their places of work.^8^

Second, to speak to a broader global audience, some companies, like Amazon, have voice-picking technology in their warehouses (Smids et al., 2020). Besides the automation of product-picking jobs in the warehouse, this technology, according to recent research, reduces the opportunities for workers to have human-to-human interactions at different Amazon stations (TKI Dinalog-Dutch Institute for Advanced Logistics, 2020). Furthermore, workers within this context hold the view that they do not experience social relations as much as they did when they had their human counterparts do the job that the voice-picking robots now do; this is because they cannot have social relationships as experienced by humans with robots (Gutelius & Theodore, 2019). These are a few ways automated technologies affect the value of relationality.

With remote work, people become socially isolated from each other. For most of the working hours, people only engage with their computers and the technologies that enable them to accentuate their work (Raghuram et al., 2019). This is because remote work makes workers’ presence in the traditional workplace redundant; workers only avail themselves virtually (Gajendran & Harrison, 2007; Iqbal et al., 2018). However, according to research done during the rise of COVID-19, workers complained of technostress and social isolation as adverse effects of remote work (Bin et al., 2021). Workers felt professionally isolated from colleagues as they were meant to communicate via online spaces, and fewer friendships were created (Carillo et al., 2021; Ipsen et al., 2021). These resulted in some workers’ stress and minimum work output (Toscano & Zappalà, 2020).

The implication of less social relationships that results from remote work and workspaces with automated technology could be advantageous to certain actors, such as business owners, as it allows them to maximise their profits. However, it makes work less meaningful for workers, especially people in sub-Saharan Africa and those of sub-Saharan African descent. This is because I contend that meaningful work, as stated earlier, allows for relationality within the sub-Saharan African context, and interpersonal relationships are important in sub-Saharan African societies. Additionally, engaging in a social relationship is one of the requirements for an individual to attain the moral status of personhood. The more an individual relates with others, the more the individual is considered a person.

The notion of relationship cuts across every sphere of the life of a sub-Saharan African, including the workspace. If an individual’s social relationship is hampered, it also affects the personhood of that individual. As previously pointed out, the workplace is a very important avenue for Africans to foster interpersonal relationships. Remote work and automated work affect relationships, as they obscure the opportunities for individuals to relate with each other. Given this challenge, it follows that relationality is also affected.

The future of work currently revolves around introducing sophisticated disruptive automated technologies and creating more opportunities for remote working environments. The implication of the nature of the future of work is that work will become less meaningful for people, especially those in sub-Saharan Africa, due to the lack or less opportunities for relationality. However, one might argue that if remote work does not allow interpersonal relationships to thrive, then individuals in sub-Saharan Africa must start creating relationships with technologies or start seeing technologies as colleagues. However, I ask if technologies can be called colleagues. What does it mean to be a colleague? What are the required criteria, and can advanced technologies meet these requirements? In what follows, I discuss this issue.

## Collegial Relationship and the Implication for AI Technologies and Other Advanced Techs

One of the most prevalent types of interpersonal relationships, especially for sub-Saharan Africans, like I have pointed out earlier, is the relationship that happens in the workplace. This is because of the considerable time we spend at our workplaces. Given the importance of work and the friendship we enjoy with those we work with, it is pertinent to understand the nature of these working relationships and to know if we can create these relationships with robots in the event that there are no human colleagues to relate with (Nyholm & Smids, 2020:2171; Betzler & Löschke, 2021:213).

In their discourse on collegial relationships, Betzler and Löschke (2021) point out three criteria (which I appraise) that a person must meet to be considered as a colleague in the workplace. First, one has to share the same status as their peers in the workplace (Betzler & Löschke, 2021: 217). This requirement implies that colleagues must be of equal status in their workplace. Second, colleagues must share the same work content and activities (Betzler & Löschke, 2021: 217). For example, a senior lecturer at a university can be colleagues with other lecturers but not with a doctoral candidate. This is because they do not share the same work status or content of work.

Finally, colleagues must have a common purpose or the same institutional affiliation (Betzler & Löschke, 2021: 217). The sameness of an institution or common purpose should not be understood only from a narrow perspective, such as working for the same company or institution. This could also be working for a vast institutional network. For example, a professor in the Faculty of Humanities at the University of Johannesburg can be a colleague of a professor in the Faculty of Engineering at the University of Pretoria. Betzler and Löschke (2021) allude that it is not always the case that all three criteria must be met for people to be considered as colleagues; however, two criteria ought to be met. For example, a medical doctor at St John’s Hospital can be a colleague to a medical doctor at Mary Slessor Hospital, as long as their interests are not in opposition. This is because they share the same work content and status, even though they share different institutional affiliations (Betzler & Löschke, 2021:218).

For Betzler and Löschke (2021), the three requirements needed for one to be called a colleague ought to produce two goods: solidarity and recognition (2021:219). First, in terms of collegial solidarity, colleagues in the workplace have good reason to be in solidarity with each other, such as offering one another assistance. For instance, suppose Diana and Thandeka work at the social media department of a company as social media consultants; both colleagues can offer each other assistance, such as covering for each other at work when the needs arise. This is because they share the sameness of status, are affiliated with the same institution, and have the sameness of work content. In this sense, they both have the ability to help each other. Second, in the aspect of collegial recognition, Betzler and Löschke (2021:221) argue that only colleagues can recognise each other’s skills, contributions, and abilities in ways that matter significantly to them. For instance, only a publishing academic colleague can understand better what it means for another colleague’s paper to get accepted or rejected by a top-rating journal.

Given the brief exposition of collegial relationships by Betzler and Löschke (2021), which I find plausible and appraise, in what follows, I ask, can high-performing and sophisticated technologies in the workplace, such as ICTs, AI, and robots be considered colleagues to humans? In the event that there are no human colleagues in the workplace in sub-Saharan Africa to meet their moral requirement of creating interpersonal relationships to be considered persons, can sub-Saharan Africans achieve this through their relationships with robots? In what follows, I briefly engage with this argument.

To address the above question, theorists like Nyholm and Smids (2020) have sought to offer arguments on how robots and high-level intelligent systems can be considered colleagues. Nyholm and Smids (2020) contend that, given the high performances of robots, especially when interacting with their human counterparts in the workplace and their behavioural patterns to help humans, it follows that they can be called colleagues (2019:2179; Ley, 2023). They argue that the current designs of robots are equipped with the capacity to support humans in different ways. To optimise their performances, we need to further equip them with robust learning techniques and capabilities (Nyholm & Smids, 2020). However, they point out that one of the limitations of these robots is their lack of inner life/subjective experience (2020:2183). They contend that the inner life of a robot is different from a human being, and this difference ought to be recognised. However, it is Nyholm and Smids’ (2020:2184) intuitions that we ought to look past the concept of subjective experiences/inner life when attributing collegial relationships to robots.

I think that the notion of subjective experience is very important in attributing the ability of collegial relationships to robots. Bringing back the goods of collegial relationship by Betzler and Löschke (2021), which I appraise, I do not see how robots and other intelligent systems can be in solidarity with their human colleagues and vice versa, through offering each other assistance. This is because robots do not have interests. A robot may assist their human colleagues when they call sick, feel tired, or have commitments, but they cannot receive such privileges from their human “colleagues.” As a result, the collegial solidarity seems to me as very instrumental and one-sided. Second, I do not think the happiness I will feel from a robot recognising my efforts at work will surpass the feeling I will get when a human colleague appreciates my work. This is because I do not think they understand the stress, pains, and joys involved in my job. These experiences can only be understood by those that have subjective experiences. Thus, robots cannot be considered human colleagues in terms of the good of collegial relationships, as espoused by Betzler and Löschke (2021).

But to drive the argument home to sub-Saharan Africa, I do not think creating friendships with robots will count as a requirement for the moral status of personhood for a human being within the context of sub-Saharan Africa. Although theorists like Christopher Wareham (2020) have attributed the status of personhood, in the sub-Saharan usage of the term, to AI, I have argued elsewhere that Wareham’s attribution of personhood to AI in the sub-Saharan African sense is implausible (Ugar, 2022). I argued that AI cannot be persons in the sub-Saharan African sense because they cannot be morally responsible or accountable for their actions, an important requirement for personhood, in some African accounts of personhood (Ikuenobe, 2016; Ugar, 2022). However, my view in this current paper is that these high-performing technologies cannot allow themselves to enjoy solidarity with humans because they lack interests. This view is also shared by Friedman (2022). Currently, because these technologies cannot be regarded as persons within the sub-Saharan African use of the term, it follows that persons within this locale do not have a moral responsibility to build friendships with them, even at their places of work, to advance their personhood. In what follows, I return to my argument on the impact of remote work on sub-Saharan Africans as I consider some possible objections.

## Possible Objections

In the fifth section, I argued that remote work and automated work are the key elements in the future of work. However, these elements result in isolation for workers, taking away opportunities for relationality. I then showed that relationality or interpersonal relationship is an important element that constitutes meaningful work in sub-Saharan Africa. Obscuring relationships by introducing automated technology and a remote working environment leads to fewer relationships in the workspace. This then results in meaninglessness in the workspace. In this section, I now engage with possible objections to my argument.

A critic may argue that remote work is a good thing on account that it provides the opportunity for workers to take care of their families (Gajendran & Harrison, 2007), balance work and family, and present employees with ample time to relate with family members. If this is the case, it follows that remote work offers workers the opportunity to create social relationships. If workers can create social relationships with their family members while working, then it means that workers can still have the opportunity to exercise their personhood within the African context. In addition, the critic may also point out that virtual working space does not mean fewer relationships, as employees can still relate with each other virtually. This follows that moving to remote work and automated work does not necessarily affect the future of work and meaningful work from a sub-Saharan African perspective.

### Response to Objections

The above objection seems to be plausible; as a result, it requires a cautionary response. First, Africans do not only consider the nuclear family to be members of a particular family. Family membership also extends to extended family members and, in some instances, friends and colleagues. Individuals are expected to create social ties beyond their nuclear family members. The workspace offers this opportunity for members to relate with each other. Relating with nuclear family members is not enough for an individual to become a person. The individual must relate with community members, including those in the workspace. Thus, the workspace is still an important place for social relationships to take place. For work to be meaningful for workers within the sub-Saharan African context, it must provide avenues for interpersonal relationships.

Second, it is true that virtual spaces are also spaces that allow for interpersonal relationships. For example, people meet on social platforms such as Zoom, Microsoft Teams, and Discord for work-related meetings and still find ways of socialising. However, the virtual space is not the same as the physical space. Furthermore, interpersonal relationship is not merely about talking to each other online; it involves having lunch together as colleagues, meeting in the kitchen for coffee, and passing by each other’s offices for a quick conversation. These are important aspects of interpersonal relationships within the African sense that are expected to happen in the workplace. In addition, these aspects enable a meaningful work environment for people of sub-Saharan Africa. The virtual space does not offer these possibilities. As a result, the virtual space, a remote work environment, is insufficient to foster interpersonal relationships and meaningful work for sub-Saharan Africans.

## Concluding Remarks

In this paper, I argued that remote work and automated technology, as important aspects of the future of work, threaten relationality. I showed the importance of relationality to meaningful work in sub-Saharan Africa. As a result, I contended that gravitating to automated technology and remote work implies gravitation to meaningless work in sub-Saharan Africa. I began this paper by providing an exposition of the concept work. I argued that it is complex to define work. However, it involves jobs that are paid for or not paid for but could be paid jobs. Furthermore, I showed that work also meant an activity that involved the exertion of energy with a specific purpose, such as economic remuneration. In the second section, I discussed what makes work meaningful. I should say that meaningful work is characterised by autonomy, social relationship, and the ability to upskill oneself. In the third section, I alluded that remote work and automated technology contribute to workplace isolation due to the lack of relationality, a key factor for meaningful work in sub-Saharan Africa.

Given that remote work and automated technology are major elements of the future of work, it follows that the future of work for sub-Saharan Africans may become meaningless. Consequently, section six considered the possibility of socially disruptive technologies being accorded the status of colleagues. However, I argued that robots and other disruptive technologies cannot be seen as colleagues in sub-Saharan Africa because they cannot be considered persons, on the one hand; on the other hand, they cannot allow themselves to enjoy the reciprocal good of a collegial relationship. I raised possible objections to my argument and responded afterwards.

Finally, it is important that I reiterate that this paper adds to the literature on meaningful work and the future of work in a disruptive technological era by discussing the effects of remote work and the automation of work to an important aspect of what constitutes meaningful work for Africans. Furthermore, more research can be done on bridging the gap between the future of work and meaningful work for Africans. In addition, researchers can also investigate the effect of remote work in other contexts, such as China, as they share similar worldviews with sub-Saharan Africans.

## Data Availability

Not applicable.

## Footnotes

1 I use the concept of “disruptive technologies” similar to Hopster’s (2021) socially disruptive technology. Disruptive technology or socially disruptive technologies are those technologies that continually distort our current traditional social norms and our engagement with the world due to their unpredictability (Hopster 2021:2–3). The constant evolution of these technologies forces us to revisit our traditional norms, such as how we relate with each other, and so on (Ugar, 2022).

2 Social technical systems are technologies that are capable of complying with human values, as well as embodying these values (van de Poel, 2020:389). These technologies have some form of agency which distinguishes them from traditional technologies, such as screwdrivers, knives, and hammers (van de Poel, 2020).

3 In this paper, I focus on sub-Saharan Africa because of the importance of the notion of relationality to the culture, worldviews, and ethics of those in this environment. For instance, in order for a human being to be considered and valued in the community, the individual must be able to relate more with the rest of the members of the community (Friedman 2022; Mbiti 1970; Metz 2022; Tutu 1999; Ugar 2022). In this sense, the notion of interpersonal relationships is a moral responsibility that ought to be achieved for one to be considered more of a person. This idea is slightly different in some Western cultures, where interpersonal relationships are a matter of choice and not moral criteria for attaining or receiving any moral recognition. What makes an individual a person is the idea that the individual is capable of making rational and autonomous choices (Molefe 2017). Given these two contrasting requirements for moral status, it then justifies my focus on sub-Saharan Africa in my discussion of the notion of relationality, as it is important to them. Finally, this is not to suggest that the rest of the world, especially those in the West, do not value the good of relationships. My claim is that even if those in the West value relationship, it is still a matter of choice and not a moral requirement for attaining excellence, and it is valued more in sub-Saharan Africa.

4 The expanded conventional definition holds that anything related to paid activities, either directly or indirectly, even though they could be unpaid, can still be considered work (Eichler and Mathews, 2004). My focus in this paper is on the conventional definition of work. This is because I am more concerned with the collegial relationship that happens in the place of work. In addition, since I am focusing on sub-Saharan Africa, I have to spell out that it is my thinking that people in sub-Saharan Africa do not just work for fun, given the region’s economic situation. They work because they have to provide for their families.

5 While this section focuses primarily on the notion of meaningful work, it also engages with the proliferation of technologies and their implication to meaningful work.

6 This point can be contested because AI can be combined with traditional work rather than substituting the latter. However, I do not engage with that argument here, as it is not my point of focus.

7 It is important that I mention that the notion of personhood in sub-Saharan Africa is not static. One can become more of person or less of a person. The more one accrues qualities that are needed for the gradation of personhood, the more the individual is graded (Ikuenobe, 2016; Menkiti, 1984). Thus, the more individual creates social relationships, the more the individual is considered a person.

8 The idea of the work relationship that exists on Teams and Zoom can be considered as a work-metaverse relationship. One might ask if the work relationship that might exist in the metaverse environments can be achieved in the real world while still counting as requirement for collegiality. I am sceptical that the collegial metaverse relationship cannot be achieved compared to the real-life working environment, at least not in the context of sub-Saharan Africa, as I have strongly shown. This is because interpersonal relationships have more value when they happen in the real world. However, further research can be done in this area to see the possibility of the metaverse relationship for Africans.

## List of Abbreviations

AI: Artificial intelligence
COVID-19: Coronavirus Disease 2019
IT: Information technology
